# Reducing Mortality and Morbidity in Children with Severe Combined Immunodeficiency in Switzerland: the Role of Newborn Screening

**DOI:** 10.1007/s10875-023-01640-2

**Published:** 2024-01-02

**Authors:** Maarja Soomann, Seraina Prader, Aline Pinto Monteiro, Ulrike Zeilhofer, Mathias Hauri-Hohl, Tayfun Güngör, Jana Pachlopnik Schmid, Johannes Trück, Matthias Felber

**Affiliations:** 1 Division of Immunology and the Children’s Research Center, University Children’s Hospital Zurich, University of Zurich, Steinwiesstrasse 75, 8032 Zurich, Switzerland; 2 Division of Stem Cell Transplantation and the Children’s Research Center, University Children’s Hospital Zurich, University of Zurich, Zurich, Switzerland

**Keywords:** SCID NBS, Survival in SCID, HSCT in SCID, SCID outcomes

## Abstract

Newborn screening (NBS) for severe combined immunodeficiency (SCID) has been introduced in various countries with the aim of reducing morbidity and mortality. However, studies analyzing outcomes before and after the implementation of NBS programs remain limited. This study sought to compare the outcomes of SCID patients identified through Switzerland’s national SCID NBS program, introduced in January 2019, with those of a historical cohort diagnosed between 2007 and 2019. The study included seven patients (32%) identified through NBS, and 15 (68%) born before NBS implementation and diagnosed based on clinical signs. Children in the NBS group were younger at diagnosis (median age 9 days vs 9 months, *P* = .002) and at hematopoietic stem cell transplantation (HSCT, median age 5 months vs 11 months, *P* = .003) compared to the clinical group. The NBS group had a lower incidence of infections before HSCT (29% vs 93%, *P* = .004). Although not statistically significant, the overall survival rate on last follow-up was higher in the NBS group (86% vs 67%, *P* = .62). Importantly, patients with active infections undergoing HSCT had a significantly lower overall survival probability compared to those without (*P* = .01). In conclusion, the introduction of NBS in Switzerland has led to earlier and often asymptomatic diagnosis of affected children, enabling timely intervention, infection prevention, and prompt treatment. These factors have contributed to higher survival rates in the NBS group. These findings underscore the critical importance of NBS for SCID, offering potential life-saving benefits through early detection and intervention.

**Supplementary Information:**

The online version contains supplementary material available at 10.1007/s10875-023-01640-2.

## Introduction

The rationale behind newborn screening (NBS) for severe combined immunodeficiency (SCID) originates from work showing higher survival in patients undergoing hematopoietic stem cell transplantation (HSCT) in the first 3.5 months of life compared with older children [1–5], especially in those who remained infection-free until transplantation [5]. Although the first NBS programs were introduced more than a decade ago [6], until very recently there was no direct evidence that NBS improves the outcome of SCID patients available [7]. The only other previous comparative study from the USA failed to show better survival in patients identified triggered by NBS or positive family history in comparison to patients diagnosed on clinical grounds [8].

The Swiss SCID NBS program was introduced nationally in January 2019 and utilizes polymerase chain reaction (PCR) to quantify T cell receptor excision circles (TREC) [9] and kappa-deleting excision circles [10] from dried blood spots with a commercially available kit (SPOT-it™ TREC & KREC Screening Kit, ImmunoIVD, Sweden). The screening algorithm involves multiple steps that distinguish between urgent positive results with TREC levels between 0 and 1 copies/punch, reduced TREC levels of 2–5 copies/punch, and isolated reduced KREC levels of 0–3 copies/punch. A more detailed description of the screening process, diagnostic testing, and infection prevention measures recommended for Swiss children with suspected SCID has been published previously [11]. In Switzerland, both NBS and HSCT for inborn errors of immunity have been centralized to a single reference center, namely the University Children’s Hospital Zurich, which serves as the referral point for all patients with suspected SCID.

The primary objective of our study was to conduct a comparative analysis between the outcomes of SCID patients diagnosed through the NBS program and a recent national historical cohort of patients born before NBS was implemented. By undertaking this comparison, we sought to gain valuable insights into the effectiveness and benefits of implementing NBS for SCID in our and similar healthcare systems.

## Methods

Data was collected retrospectively from electronic health records. All patients fulfilling ESID criteria [12] for either SCID or atypical SCID, diagnosed from the implementation of electronic laboratory records in 2007 until the end of 2020, and diagnosed at or referred to our institution as the only center for HSCT for inborn errors of immunity in Switzerland, were included. All patients born since the introduction of the national screening program were classified into the NBS group, while those born prior to its inception were categorized into the clinical group. Table 1S provides definitions and classifications used in this work [13–15].

In the NBS group, all patients underwent whole exome sequencing. In the clinical cohort, the approach to genetic testing varied: most patients had a small number of individual genes sequenced, targeting those most likely to be affected based on their phenotype and the available data at the time, while a minority underwent whole exome sequencing.

Categorical variables were compared using Fisher’s exact test and continuous variables using the Mann–Whitney *U*-test. We conducted a survival analysis with survival time defined as the time from first HSCT to death with survivors censored on last documented clinical follow-up using the Kaplan–Meier estimator, Cox regression, and log-rank test. Data was analyzed with R version 4.1.3 [16], packages ggthemes, knitr, survminer, survival, tidyquant, and tidyverse.

The study was granted permission by the Cantonal Ethics Commission of Zurich (2022–01029). Parents or legal guardians gave informed consent per agreed protocol.

## Results

A total of 22 patients were diagnosed with SCID or atypical SCID between 2007 and 2020. All underwent HSCT and were included in the analysis. Among these patients, one-third (7) were born after the implementation of NBS (NBS group), while the remaining two-thirds (15) were born earlier and received diagnoses triggered by their clinical presentation or family history (clinical group). All patients born since the introduction of the national NBS program were diagnosed prompted by NBS. The last patient diagnosed solely through clinical means was born in 2018 but diagnosed and transplanted in 2019. Patients in the clinical group were thus diagnosed and transplanted between 2007 and 2019, while patients identified through NBS received their diagnosis and treatment between 2019 and 2021. Using historical birth data [17], it can be estimated that the incidence of SCID was approximately 1 in 66,000 live births before the implementation of screening (2007 to 2018), and it increased to approximately 1 in 25,000 during the first 2 years of screening (2019 to 2020).

Six patients were female and 16 were male. The age at diagnosis was significantly lower in the NBS group compared to the clinical group (median 9 days vs 9 months, *P* = 0.002). For patients diagnosed through NBS, the median time from heel prick to diagnosis was 5 days (range, 0 to 10 days). None of the patients in the NBS group had a relevant positive family history.

On their first immunological review (median age 9; range 4 to 13 days), all patients diagnosed through NBS were free of infection. Nonetheless, two patients, aged 4 and 10 days, respectively, already had a generalized rash, marking their first sign of Omenn syndrome, which was subsequently confirmed through further diagnostic evaluation in both cases. In contrast, most patients in the clinical group initially presented with infections (9, 60%). In one patient, their initial infection (*Pneumocystis jirovecii* pneumonia at 4 months of age) required intensive care treatment. Among four other patients, their first episodes involved lower respiratory and gastrointestinal tract infections, requiring hospitalization on regular wards, with onset between 2 weeks and 3 months of age. An additional four patients initially managed their respiratory and gastrointestinal infections in an outpatient setting before experiencing more severe episodes that required hospitalization. A smaller number of patients showed signs of autoimmunity or failure to thrive as their first symptoms of immunodeficiency, with 2 cases each. Only 1 patient in the clinical group was diagnosed based on a positive family history. The median time from initial presentation of symptoms to diagnosis in the clinical group was 4 months (range 3 days to 8 years). A comparison between the two groups, including their general characteristics, initial presentation, and categorization of the underlying disease, is detailed in Table 1.

**Table 1:** General characteristics of the study population and comparison of the newborn screening and clinical group based on initial presentation and type of underlying disease

	All (*n* = 22)	NBS (*n* = 7)	Clinical (*n* = 15)	*P* value
Sex, *n*				.12
Male	16	7	9	
Female	6	0	6	
Initial presentation, *n*				
NBS	7	7*	n/a	
Infection	9	0	9	
Autoimmunity	2	0	2	
Failure to thrive	2	0	2	
Family history	1	0	1	
Generalized rash	1	0	1	
Age at diagnosis, median (minimum to maximum)	5 months (3 days to 13 years)	9 days (4 to 13 days)	9 months (3 days to 13 years)	.002
Type of SCID, *n*				.66
T–B–NK–	1	1	0	
T–B–NK +	9	3	6	
T–B + NK–	5	1	4	
T–B + NK +	7	2	5	
Underlying defect, *n*				.47
RAG1	5	3	2	
RAG2	1	0	1	
ADA	2	1	1	
LIG4	1	0	1	
NHEJ1	1	0	1	
IL2RG	6	1	5	
IL7R	3	0	3	
RMRP	2	1	1	
JAK3	1	1	0	

*ADA*, adenosine deaminase; *IL2RG*, interleukin 2 receptor gamma; *IL7R*, interleukin 7 receptor alpha chain; *JAK3*, Janus kinase 3; *LIG4*, DNA ligase 4; *n/a*, not applicable; *NBS*, newborn screening; *NHEJ1*, nonhomologous end-joining factor 1; *RAG1*, recombination activating gene 1; *RAG2*, recombination activating gene 2; *RMRP*, RNA component of mitochondrial RNA-processing endoribonuclease; *SCID*, severe combined immunodeficiency. *Two patients had symptoms of Omenn syndrome at their first immunological review

Mutations in either RAG1 or RAG2 were identified in a quarter of all patients (6), with an equal number of patients having mutations in IL2RG (6). The proportion of patients with RAG1/2 mutations was higher in the NBS group compared to the clinical group (43% vs 20%, *P* = 0.33). All patients with RAG1/2 mutations had distinct compound heterozygous mutations. Only 19% of all patients with autosomal recessive disorders had homozygous mutations. Further details on identified mutations can be found in Table 2.

**Table 2:** Mutation details

Gene	Patient presentation	Zygosity	Transcript	DNA sequence mutation	Protein sequence change
ADA	Clinical	Compound heterozygous	n/r	c.646G > A c.838_841delGTCA	p.Gly216Arg p.Ala278fs*29
ADA	NBS	Compound heterozygous	NM_000022.3	c.218 + 1G > A c.489dup	n/r p.Lys164Glnfs*7
IL2RG	Clinical	Hemizygous	n/r	c.268 T > C	p.Leu85Pro
IL2RG	Clinical	Hemizygous	n/r	c.591_601delCAAGGAACAAT	n/r
IL2RG	Clinical	Hemizygous	n/r	c.591_601delCAAGGAACAAT	n/r
IL2RG	Clinical	Hemizygous	n/r	n/r	p.Tyr89Cys
IL2RG	Clinical	Hemizygous	NM_000206.2	c.41 T > C	p.Leu14Pro
IL2RG	NBS	Hemizygous	NM_000206.2	c.322 T > C	p.Ser108Pro
IL7R	Clinical	Compound heterozygous	NM_002185.3	c.616C > T c.617G > A	p.Arg206* p.Arg206Gln
IL7R	Clinical	Homozygous	n/r	c.353G > A	p.Cys118Tyr
IL7R	Clinical	Homozygous	n/r	n/r	n/r
JAK3	NBS	Compound heterozygous	NM_000215.3	c.385del c.2647A > C	p.Asp130Thrfs*17 p.lle883Leu
LIG4	Clinical	Compound heterozygous	n/r	c.877C > T c.1345A > C	n/r
NHEJ	Clinical	Homozygous	NM_024782.2	c.506A > T	p.Glu169Val
RAG1	Clinical	Compound heterozygous	n/r	c.1794C > A c.C2387T	p.Arg561His p.Arg759Cys
RAG1	NBS	Compound heterozygous	NM_000448.2	c.1331C > T c.2018_2025del	p.Ala444Val p.Ala673Glufs*6
RAG1	NBS	Compound heterozygous	NM_000448.2	c.256_257del c.2488A > T	p.Lys86Valfs*33 p.Lys830*s
RAG1	NBS	Compound heterozygous	NM_000448.2	c.413del c.2487_2488delinsTT	p.Leu138Glnfs*26 p.Arg829_Lys830delinsSer*
RAG1	Clinical	Compound heterozygous	NM_000448.2	c.535dup c.2918G > A	p.Cys179Leufs*10 p.Arg973His
RAG2	Clinical	Compound heterozygous	NM_000536.2	c.475C > T c.1352G > C	p.Arg159Cys p.Gly451Ala
RMRP	Clinical	Compound heterozygous	n/r	c.4C > T c.35C > T	n/a
RMRP	NBS	Compound heterozygous	NR_003051.3	c.5C > T c.-24_-12dup	n/a

*ADA*, adenosine deaminase; *IL2RG*, interleukin 2 receptor gamma; *IL7R*, interleukin 7 receptor alpha chain; *JAK3*, Janus kinase 3; *LIG4*, DNA ligase 4; *n/a*, not applicable; *NBS*, newborn screening; *NHEJ1*, nonhomologous end-joining factor 1; *n/r*, not reported; *RAG1*, recombination activating gene 1; *RAG2*, recombination activating gene 2; *RMRP*, RNA component of mitochondrial RNA-processing endoribonuclease

The median time from diagnosis to HSCT was 4 months and was similar in both groups (*P* = 0.46, Table 3). However, the median age at HSCT was significantly lower in the NBS compared to the clinical group (5 vs 11 months, *P* = 0.003). Furthermore, patients in the NBS group were significantly less likely to acquire infections prior to HSCT (29% vs 93%, *P* = 0.004). Within the NBS group, one patient contracted a respiratory syncytial virus (RSV) infection before palivizumab could be started (a detailed case description has been previously published [18]). In the second patient, routine infection screening detected CMV viremia at the age of 24 days. This patient received treatment with ganciclovir/valganciclovir for a duration of 2 months, until their blood CMV levels became undetectable. Subsequently, prophylactic valganciclovir was continued, and the patient remained asymptomatic.

**Table 3:** Comparison of complications and transplantation characteristics in the newborn screening and the clinical group

	All (*n* = 22)	NBS (*n* = 7)	Clinical (*n* = 15)	*P* value
Infections, *n*				
Pre-HSCT	16	2	14	.004
Active undergoing HSCT	7	1	6	.35
1st year post-HSCT	17	4	13	.27
Omenn syndrome, *n*	3	3	0	.03
Autoimmunity, *n*				
Pre-HSCT	6	0	6	.12
AIHA	2	0	2	
ITP	2	0	2	
Enteropathy	3	0	3	
Vitiligo	2	0	2	
Myocarditis	1	0	1	
Post-HSCT	7	2	5	.99
AIHA	3	1	2	
Encephalitis	1	0	1	
Glomerulonephritis	2	0	2	
Thyroiditis	3	1	2	
Vitiligo	1	1	0	
Time from diagnosis to HSCT, median (minimum to maximum)	4 months (22 days to 4 years)	4 months (3 to 8 months)	4 months (22 days to 4 years)	.46
Age at HSCT, median (minimum to maximum)	10 months (3 months to 17 years)	5 months (4 to 8 months)	11 months (3 months to 17 years)	.003
Donor type, *n*				.12
MRD	3	0	3	
MUD	11	6	5	
MMUD	5	0	5	
MMRD	3	1	2	
Conditioning regimen, *n*				.42
Serotherapy/none	3	0	3	
RIC/RTC	17	7	10	
Busulfan-based	13	6	7	
Median total AUC	64 mg/L × h	64 mg/L × h	63 mg/L × h^‡^	
Cyclophosphamide	2	0	2	
Treosulfan	2	1	1	
MAC	2	0	2	
Median stem cell dose (CD34 + cells/kg), median (minimum to maximum)	7.6 × 10^6^ (4.2 × 10^5^ to 1 × 10^8^)	51 × 10^6^ (2.2 × 10^6^ to 1 × 10^8^)	3.5 × 10^6^ (4.2 × 10^5^ to 3.3 × 10^7^)	.06
GvHD prophylaxis, *n*				.10
CsA	5	0	5	
CsA + MMF	16	6	10	
CsA + MMF + sirolimus	1	1	0	
Complications, *n*				
Graft failure	4	0	4	.26
VOD	4	0	4	.26
aGvHD	9	2	7	.65
cGvHD	3	1	2	> .99
Myeloid chimerism at last follow-up, *n*				.82
< 2%	2	0	2	
2 to 10%	3	1	2	
10 to 90%	5	3	2	
≥ 90%	11	3	8	
n/a	1	0	1	
Secondary procedures, *n*				.67
Stem cell boost	1	0	1	
Second HSCT	3	0	3	
None	18	7	11	

^‡^Data available on five of seven patients. *aGvHD*, acute graft-versus-host disease; *AIHA*, autoimmune hemolytic anemia; *AUC*, area under the curve; *CsA*, ciclosporin A; *cGvHD*, chronic graft-versus-host disease; *GvHD*, graft-versus-host disease; *HSCT*, hematopoietic stem cell transplantation; *ITP*, immune thrombocytopenia; *NBS*, newborn screening; *MAC*, myeloablative conditioning; *MMF*, mycophenolate mofetil; *MMUD*, mismatched unrelated donor; *MMRD*, mismatched related donor; *MRD*, matched related donor; *MUD*, matched unrelated donor; *n/a*, data not available; *NBS*, newborn screening; *RIC*, reduced intensity conditioning; *RTC*, reduced toxicity conditioning; *VOD*, hepatic veno-occlusive disease

**Fig. 1:**
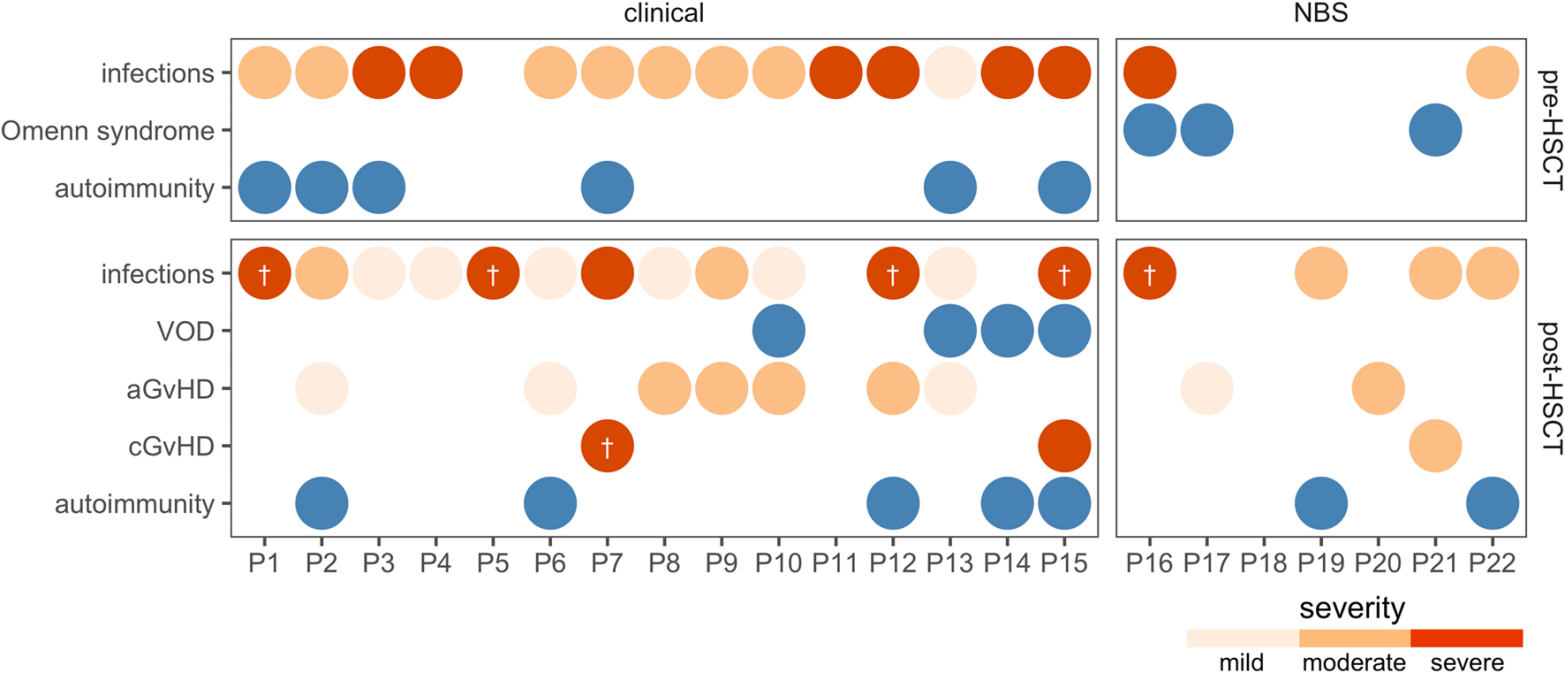
Constellation of complications in individual patients of the clinical and the NBS groups before and after hematopoietic stem cell transplantation (HSCT). Infection and graft-versus-host disease (GvHD) severity are presented on a three-step scale. White crosses mark the causes of death in the deceased. Blue circles mark the presence of complications. aGvHD, acute graft-versus-host disease; cGvHD, chronic graft-versus-host disease; HSCT, hematopoietic stem cell transplantation; NBS, newborn screening; VOD, hepatic veno-occlusive disease

In total, nearly half of the patients in the NBS group developed Omenn syndrome (all had mutations in *RAG1*), whereas there were no such cases in the clinical group (43% vs 0%, *P* = 0.03). None of the patients in the NBS group developed other forms of autoimmunity before undergoing HSCT, while these conditions were relatively prevalent in the clinical group (0% vs 40%, *P* = 0.12). An overview of complications in individual patients, including the severity of infections and autoimmunity before and after HSCT in both groups, can be found in Fig. 1.

Although none of the patients in the NBS group had a matched related donor accessible for HSCT, matched unrelated donors could be found for the majority of these patients (86%, Table 3). In the clinical group, one-fifth received HSCT from a matched related donor. Mismatched donors had to be used in nearly half of the clinical group cases. At the time of the last follow-up, none of the patients in the NBS group had required a second transplant or stem cell boost, while this was the case for 4 patients (27%) in the clinical group. Further details on transplant characteristics, including donor types, conditioning regimens, graft-versus-host disease (GvHD) prophylaxis and stem cell dose, and myeloid chimerism as well as HSCT-related complications, can be found in Table 3.

**Fig. 2:**
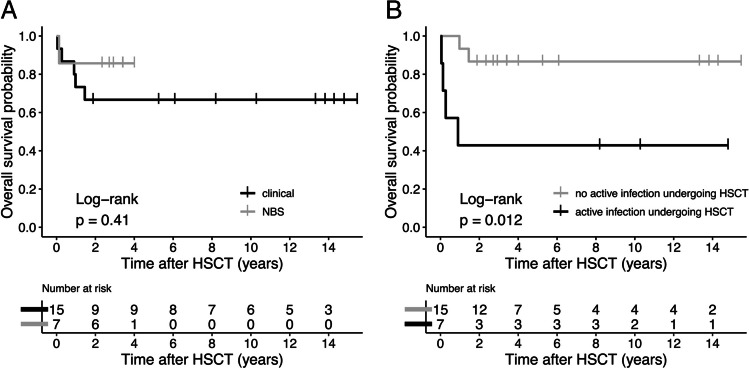
Overall survival probability in patients identified through newborn screening compared to clinically diagnosed patients (**A**) and in patients with an active infection in comparison to patients without an active infection undergoing hematopoietic stem cell transplantation (HSCT) (**B**)

Total follow-up time in survivors ranged from 23 months to 15 years post-HSCT. All six deaths occurred within the first 18 months after HSCT (Fig. 2). Infections were the most common cause of mortality (83%). Fatal infections in the clinical group included enteroviral meningoencephalitis, systemic adenovirus infection combined with systemic cytomegalovirus infection, and Aspergillus and Pseudomonas sepsis. Additionally, one patient in the clinical group died of chronic grade III intestinal GvHD [19]. In the NBS group, the single deceased patient died due to RSV pneumonia [18]. Details of the etiology of infections before, during, and in the first year after HSCT are given in Table 2S.

Overall survival on last follow-up was higher in the NBS group compared to the clinical group, although this difference was not statistically significant (86% vs 67%, *P* = 0.62). Survival analysis also showed a higher overall survival probability in the NBS group, but the difference was not statistically significant (HR 0.41, 95% CI 0.05 to 3.55, *P* = 0.42, Fig. 2A). Overall probability of survival was significantly lower in patients with active infection undergoing HSCT (HR 6.80, 95% CI 1.22 to 37.82, *P* = 0.03, Fig. 2B). There was no significant impact of pre-HSCT autoimmunity, graft source, conditioning regimen, or HLA-match on overall survival probability (Fig. 1S).

No significant difference in the rate of acute or chronic GvHD was observed between the two groups (Table 3, Fig. 1). Patients identified through NBS demonstrated a higher likelihood of achieving platelet engraftment by day 30 post-HSCT than the clinical group (100% vs 42%, *P* = 0.03). However, no significant differences were found between the groups in terms of neutrophil engraftment and the time from HSCT or immune reconstitution (Fig. 2S). Figure 3S illustrates the course of CD3 + , CD4 + , naïve CD4 + lymphocyte counts, and recent thymic emigrant counts before and after HSCT in both groups. Notably, there were four cases of graft failure, all in the clinical group. Among these cases, two were attributed to active infection and inflammation during HSCT, one to intensified immunosuppression in the context of autoimmunity and GvHD, and one was due to insufficient cell numbers in the transfused product (further details available in Table 3S). All 4 cases of hepatic veno-occlusive disease (VOD) occurred in the clinical group. Defibrotide was administered to all patients, and all recovered.

The donor chimerism in lymphocyte subsets among survivors at their last follow-up was similar between the NBS and the clinical groups (Fig. 4S). It was possible to discontinue immunoglobulin replacement in 81% of the survivors, with no significant difference between the groups (83% in the NBS and 80% in the clinical group). For those who remained on replacement at the last follow-up, CD19 + donor chimerism ranged from 12 to 100%.

## Discussion

Our study represents a significant contribution as the first national comparative analysis of outcomes in SCID patients diagnosed through either NBS or clinical signs in Europe. We observed that patients in the NBS group were diagnosed and underwent HSCT at a younger age compared to the clinical group, and they had a significantly lower incidence of infections. Notably, active infection during HSCT was associated with a lower probability of survival in our cohort, consistent with previous research [5, 8]. Consequently, there was a higher survival rate in the NBS compared to the clinical group, although this difference was not statistically significant. Our findings are consistent with previous studies that reported survival rates of 40–70% in clinically diagnosed patients [1, 4, 20] and rates of 83–100% in various NBS cohorts [7, 8, 21–24].

The relatively small number of patients in our study, influenced by the size of our country, may have contributed to the lack of statistical significance in the difference in survival rates between the NBS and clinical groups. A recent registry-based study from North America showed, for the first time, better survival in patients detected through NBS [7]. This study included a larger cohort including 130 patients identified through NBS and over 500 patients diagnosed by clinical means. In another study, comparing the outcomes of 60 patients diagnosed through NBS or based on family history to 40 clinically identified patients did not show statistically different survival rates, similarly to our study [8].

Interestingly, the fraction of patients with mutations in RAG1/2 was notably high in our NBS group, even compared to other studies that have demonstrated higher fractions of such patients in NBS cohorts than in previous clinical cohorts [7, 25]. None of these patients had a positive family history, and all had different compound heterozygous mutations. This suggests that NBS might be detecting a significant number of patients who would otherwise remain undiagnosed and could pass away without recognition of their underlying disease. Furthermore, the much higher number of SCID patients per year in Switzerland since the introduction of NBS implies that a considerable number of children might have died without a diagnosis in the past, potentially leading to an underestimation of mortality in the clinical group. In a small country, the incidence of such a rare disease calculated solely based on the data of 2 years (NBS) may however not be representative of a longer time interval.

Our study revealed that a positive family history was present in less than 5% of the patients in our whole cohort, and 0% in our NBS cohort. This is considerably lower than the approximately 10–20% reported in larger NBS cohorts [7, 23, 24] and emphasizes that increasing awareness of the risk of reoccurrence as an alternative to NBS is not a practical option for our population.

Infections were identified as the main cause of mortality in our patients, both in the NBS and the clinical group. Notably, the incidence of pre-HSCT infections in our NBS group was markedly lower than in our clinical group and other previous studies [7, 24, 26]. Nevertheless, it is important to highlight that two patients within the NBS group did contract infections before HSCT. One of the cases involves a patient who contracted RSV before commencing standard prophylaxis with palivizumab [18]. The second patient contracted cytomegalovirus, with a positive screening PCR on the 24th day of life, potentially transmitted from his seropositive parents, despite the mother having discontinued breastfeeding. Our data underscore the significance of infection prevention and proactive infection screening, which is crucial for better outcomes in SCID patients. We demonstrate that successful infection prevention is feasible in most patients identified through NBS.

Our study stands out as the first comparative study of SCID outcomes in children diagnosed through NBS compared to clinical illness, covering an entire country in Europe. Although the higher probability of overall survival in the NBS group did not reach statistical significance, it was considerably higher compared to the clinical group. Our findings highlight that successful infection prevention can be achieved in patients identified through NBS, ultimately leading to considerably better outcomes in SCID patients.

### Supplementary Information

Below is the link to the electronic supplementary material.

Supplementary file1 (DOCX 1180 KB)

## Data Availability

The datasets generated during and analyzed during the current study are available from the corresponding author on reasonable request.
