# Supplementary material for: Reducing Mortality and Morbidity in Children with Severe Combined Immunodeficiency in Switzerland: the Role of Newborn Screening
Source: J Clin Immunol. 2024 Jan 2;44(1):39. doi: 10.1007/s10875-023-01640-2 (PMC10761526; doi:10.1007/s10875-023-01640-2)
Supplement: Supplementary file 1 — Supplementary file1 (DOCX 1180 KB) [file 10875_2023_1640_MOESM1_ESM.docx]

**Table 1S**. Definitions and classifications

| **Term** | | **Definition or classification** | |
| --- | --- | --- | --- |
| Time of diagnosis | | time of clinical diagnosis as recorded in the patient’s medical records | |
| Infection | | | |
|  | Minor | either not leading to a medical contact, not requiring treatment, or successfully treated in an outpatient setting | |
|  | Moderate | either requiring recurrent treatment in the outpatient setting or hospitalization | |
|  | Severe | either requiring intensive care or a lethal infection | |
|  | Pre-HSCT | symptoms ceased and therapy stopped before starting conditioning | |
|  | Active while undergoing HSCT | either ongoing symptoms, ongoing antimicrobial treatment for an infection or a new symptomatic infection or viremia during either conditioning or the first three days after donor cell infusion | |
|  | Post-HSCT | new symptoms or a new viremia detected later than three days after donor cell infusion | |
| Omenn syndrome | | concurrent generalized rash, elevated eosinophil count and/or immunoglobulin E level, T-cell oligoclonality on spectratyping and exclusion of maternal engraftment | |
| Autoimmunity | | diagnosis of an autoimmune disease requiring either immunosuppressive therapy or in the case of autoimmune thyroiditis hormonal substitution | |
| Conditioning regimens | | | |
|  | Reduced intensity conditioning (RIC) / reduced toxicity conditioning (RTC) | N = 13 | Fludarabine 180 mg/m^2^  Busulfan with a cumulative area under the curve (AUC) below 80 mg×h/L  Alemtuzumab 0.6 mg/kg |
|  |  | N = 2 | Fludarabine 180 mg/m^2^  Treosulfan 30 to 42 g/m^2^  ± Alemtuzumab 0.6 mg/kg |
|  |  | N = 2 | Fludarabine 120 to 150 mg/m^2^  Cyclophosphamide 40 to 60 mg/kg  Alemtuzumab 1.25 mg/kg or anti-thymocyte globulin (ATG) 7.5 mg/kg |
|  | Myeloablative conditioning (MAC) | Fludarabine 180 mg/m^2^  Busulfan AUC of 80 mg×h/L or greater  ATG 7.5 mg/kg | |
|  | Serotherapy only | no chemotherapy, only one or more antibody infusions targeting immune cells were intentionally used for conditioning prior to HSCT | |
|  | None | none | |
| Engraftment | | | |
|  | Platelet | first day of three consecutive days, with platelet counts ≥ 50×10^9^/L in peripheral blood | |
|  | Neutrophil | first day of three consecutive days, with neutrophilic granulocyte counts ≥ 0.5×10^9^/L in peripheral blood | |
| Graft-versus-host disease (GvHD) | | | |
|  | Acute GvHD | Gluckberg’s criteria^*,#^ | |
|  | Chronic GvHD | National Institutes of Health criteria^†^ | |

^*^Glucksberg H, Storb R, Fefer A, Buckner CD, Neiman PE, Clift RA, et al. Clinical manifestations of graft-versus-host disease in human recipients of marrow from HLA-matched sibling donors. Transplantation. 1974 Oct;18(4):295–304.

^#^Rowlings PA, Przepiorka D, Klein JP, Gale RP, Passweg JR, Henslee-Downey PJ, et al. IBMTR Severity Index for grading acute graft-versus-host disease: retrospective comparison with Glucksberg grade. Br J Haematol. 1997 Jun;97(4):855–64.

^†^Jagasia MH, Greinix HT, Arora M, Williams KM, Wolff D, Cowen EW, et al. National Institutes of Health Consensus Development Project on Criteria for Clinical Trials in Chronic Graft-versus-Host Disease: I. The 2014 Diagnosis and Staging Working Group report. Biol Blood Marrow Transplant. 2015 Mar;21(3):389-401.e1.

**Table 2S.** Causative organisms

|  | |  | |  | **NBS group (7 patients)** | | | **Clinical group (15 patients)** | | | |
| --- | --- | --- | --- | --- | --- | --- | --- | --- | --- | --- | --- |
|  | |  | |  | **Pre-HSCT** | **Active undergoing HSCT** | **1^st^ year post-HSCT** | **Pre-HSCT** | **Active undergoing HSCT** | | **1^st^ year post-HSCT** |
| Viruses | | | | | | | |  |  |  | |
|  | Respiratory viruses | | | |  |  |  |  |  |  | |
|  |  | | | Rhinovirus | 0 | 0 | 0 | 4 | 0 | 2 | |
|  |  | | | Parainfluenza III | 0 | 0 | 0 | 2 | 1 | 0 | |
|  |  | | | Influenza A | 0 | 0 | 0 | 1 | 1 | 0 | |
|  |  | | | Picornavirus | 0 | 0 | 0 | 1 | 0 | 0 | |
|  |  | | | Respiratory syncytial virus | 1 | 1 | 1 | 2 | 0 | 0 | |
|  | Herpesviruses | | | |  |  |  |  |  |  | |
|  |  | | | Cytomegalovirus | 1 | 0 | 1 | 2 | 1 | 1 | |
|  |  | | | Varicella-zoster virus | 0 | 0 | 0 | 1 | 0 | 0 | |
|  |  | | | Epstein-Barr virus | 0 | 0 | 0 | 0 | 0 | 1 | |
|  |  | | | Human herpesvirus 6 | 0 | 0 | 0 | 0 | 0 | 2 | |
|  | Other viruses | | | |  |  |  |  |  |  | |
|  |  | | Adenovirus | | 0 | 0 | 0 | 1 | 0 | 1 | |
|  |  | | Enterovirus | | 0 | 0 | 0 | 2 | 1 | 3 | |
|  |  | | Rotavirus | | 0 | 0 | 0 | 1 | 1 | 2 | |
|  |  | | BK | | 0 | 0 | 0 | 0 | 0 | 1 | |
| Fungi | | | | | | | |  |  |  | |
|  | | Pneumocystis jirovecii | | | 0 | 0 | 0 | 4 | 0 | 0 | |
|  | | Candida | | | 0 | 0 | 0 | 2 | 0 | 1 | |
|  | | Aspergillus | | | 0 | 0 | 0 | 0 | 0 | 2 | |
| Bacteria | | | | | | | |  |  |  | |
|  | | Staphylococcus | | | 0 | 0 | 2 | 2 | 0 | 4 | |
|  | | Gram negative | | | 0 | 0 | 0 | 6 | 0 | 4 | |
|  | | Presumed bacterial | | | 0 | 0 | 2 | 9 | 1 | 6 | |
|  | | Bacillus Calmette-Guérin | | | 0 | 0 | 0 | 1 | 1 | 0 | |

NBS – newborn screening; HSCT – hematopoietic stem cell transplantation

**Table 3S.** Details on cases with graft failure

| **Underlying defect** | **Time**  **post-HSCT** | **Type** | **Probable cause** | **Intervention** | **Outcome** |
| --- | --- | --- | --- | --- | --- |
| RAG1 | 4 months | secondary | multifactorial:  - autoimmunity (autoimmune hemolytic anemia, immunthrombocytopenia)  - graft versus host disease  - immunosuppression  - MRD with heterozygous RAG1 mutation | second procedure refused by parents | death due to Pseudomonas infection |
| RAG2 | 1 month | primary | insufficient stem cells in cord blood unit due to leak while thawing | second HSCT from MMRD 1 month post-HSCT | alive |
| IL7R | 3 months | primary | multifactorial:  - severe inflammatory reaction to alemtuzumab during conditioning  - early CMV reactivation, day +12 | stem cell boost  4 months post-HSCT | alive |
| RAG1 | 1 months | primary | multifactorial:  - relatively mild conditioning (only Fludarabine and Treosulfan)  - active CMV infection during HSCT  - active inflammation during HSCT | second HSCT from MMRD 1 month,  donor-derived ADV-specific T cell infusion 3 months post-HSCT | death due to ADV and CMV infection |

ADV – adenovirus; CMV – cytomegalovirus; HSCT – hematopoietic stem cell transplantation; IL7R – interleukin 7 receptor alpha chain; RAG1 – recombination activating gene 1; RAG2 – recombination activating gene 2; MMRD – mismatched related donor; M – matched related donor


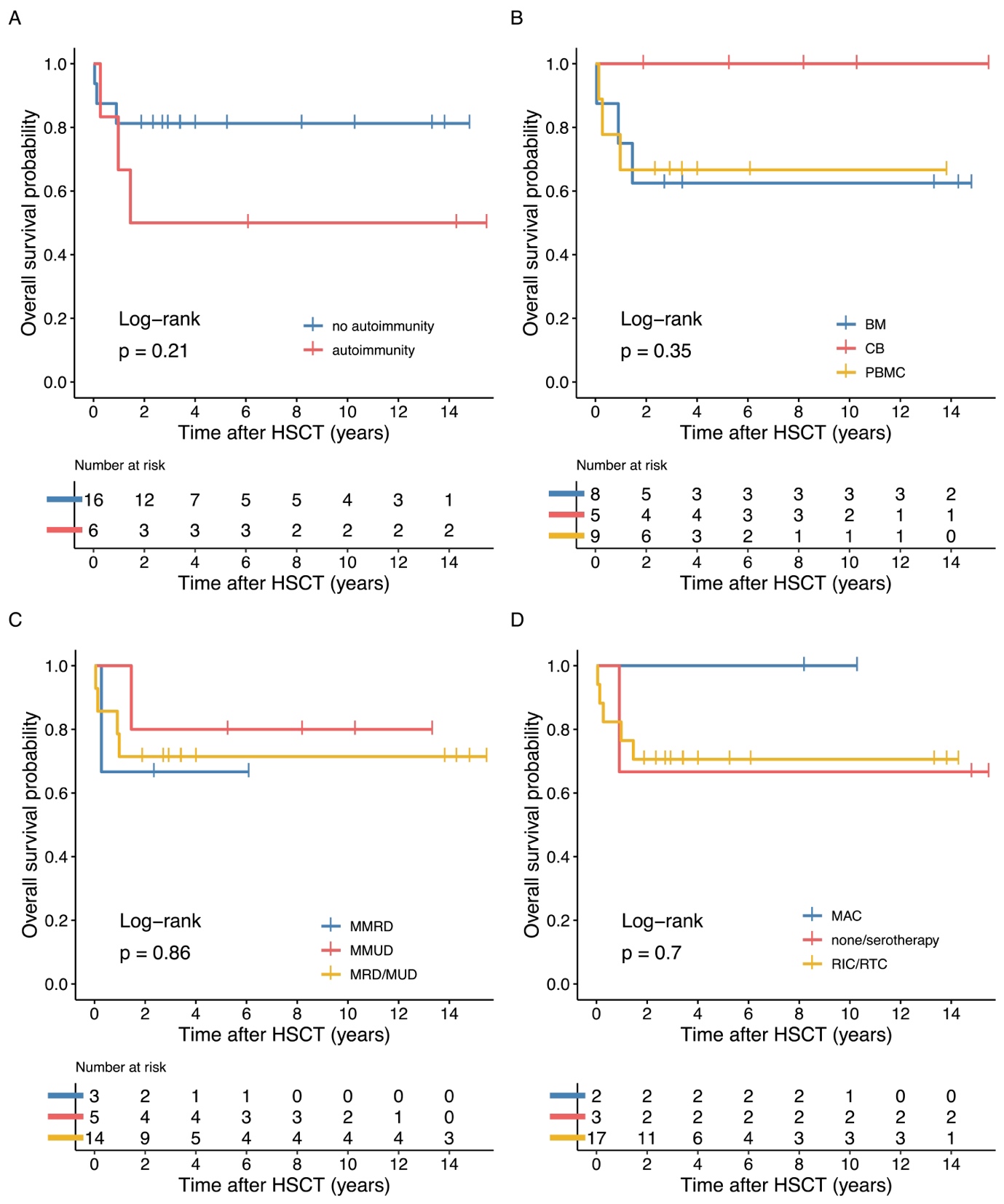


Figure 1S. Stratified survival analysis. Overall survival probability in patients with and without autoimmunity prior to transplantation (A), according to graft source (B), to HLA-match (C) and conditioning regimen (D).

BM – bone marrow; CB – cord blood; HSCT – hematopoietic stem cell transplantation; MAC – myeloablative conditioning; MMRD – mismatched related donor; MMUD – mismatched unrelated donor; MRD – matched related donor; MUD – matched unrelated donor; PBMC – peripheral blood mononuclear cells; RIC – reduced intensity conditioning; RTC – reduced toxicity conditioning


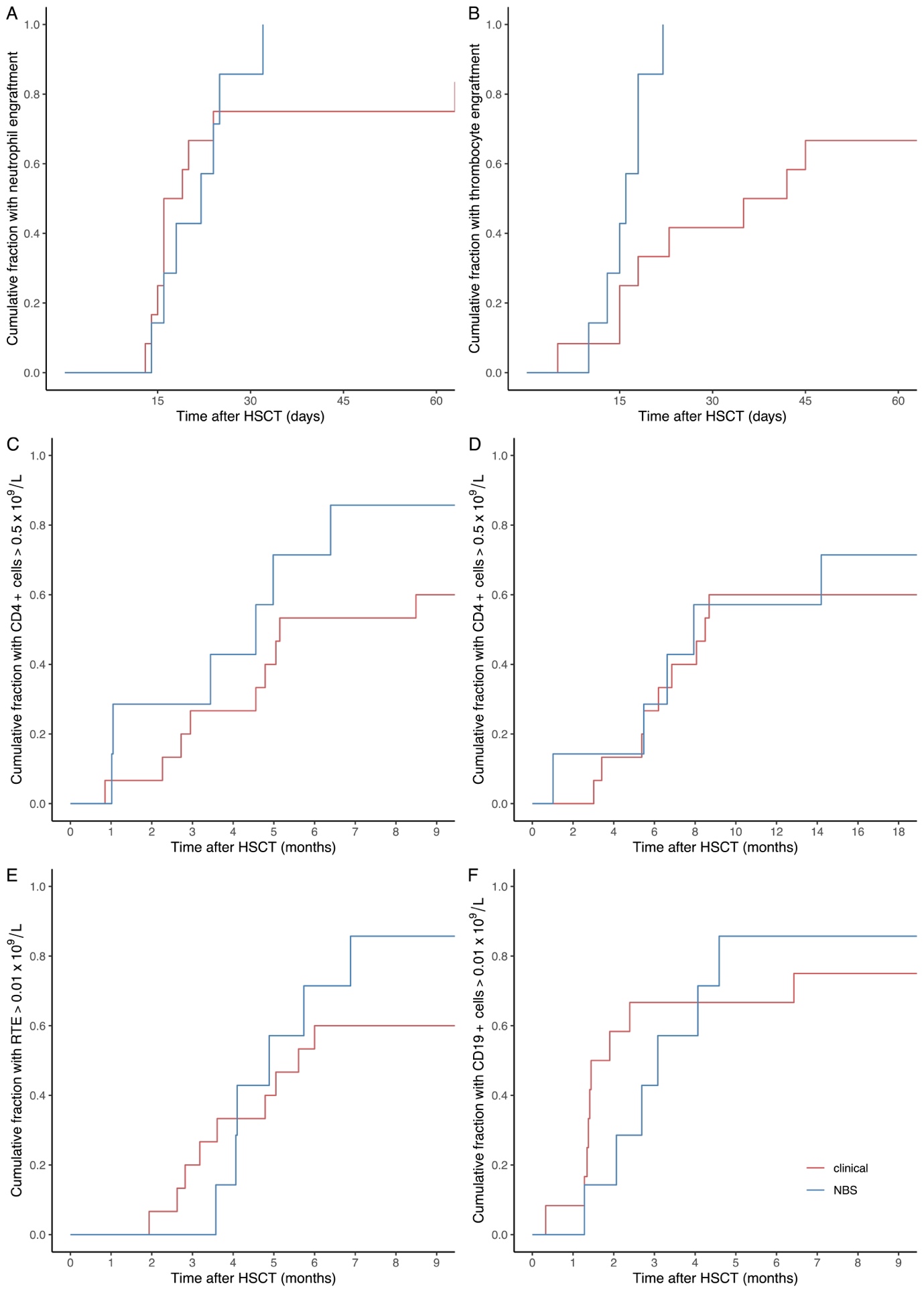
Figure 2S. Speed of neutrophil (A) and thrombocyte (B) engraftment in the newborn screening and clinically diagnosed group as cumulative fraction of patients engrafted by a point in time post-transplantation of those who underwent myeloablative or reduced intensity conditioning in each group. Speed of reaching CD4+ counts >0.2× 10^9^/L (C), CD4+ >0.5×10^9^/L (D), recent thymic emigrant (RTE) >0.01×10^9^/L (E) in all patients and for CD19+ counts >0.01×10^9^/L (F) in those who underwent myeloablative or reduced intensity conditioning. HSCT – hematopoietic stem cell transplantation; NBS – newborn screening.


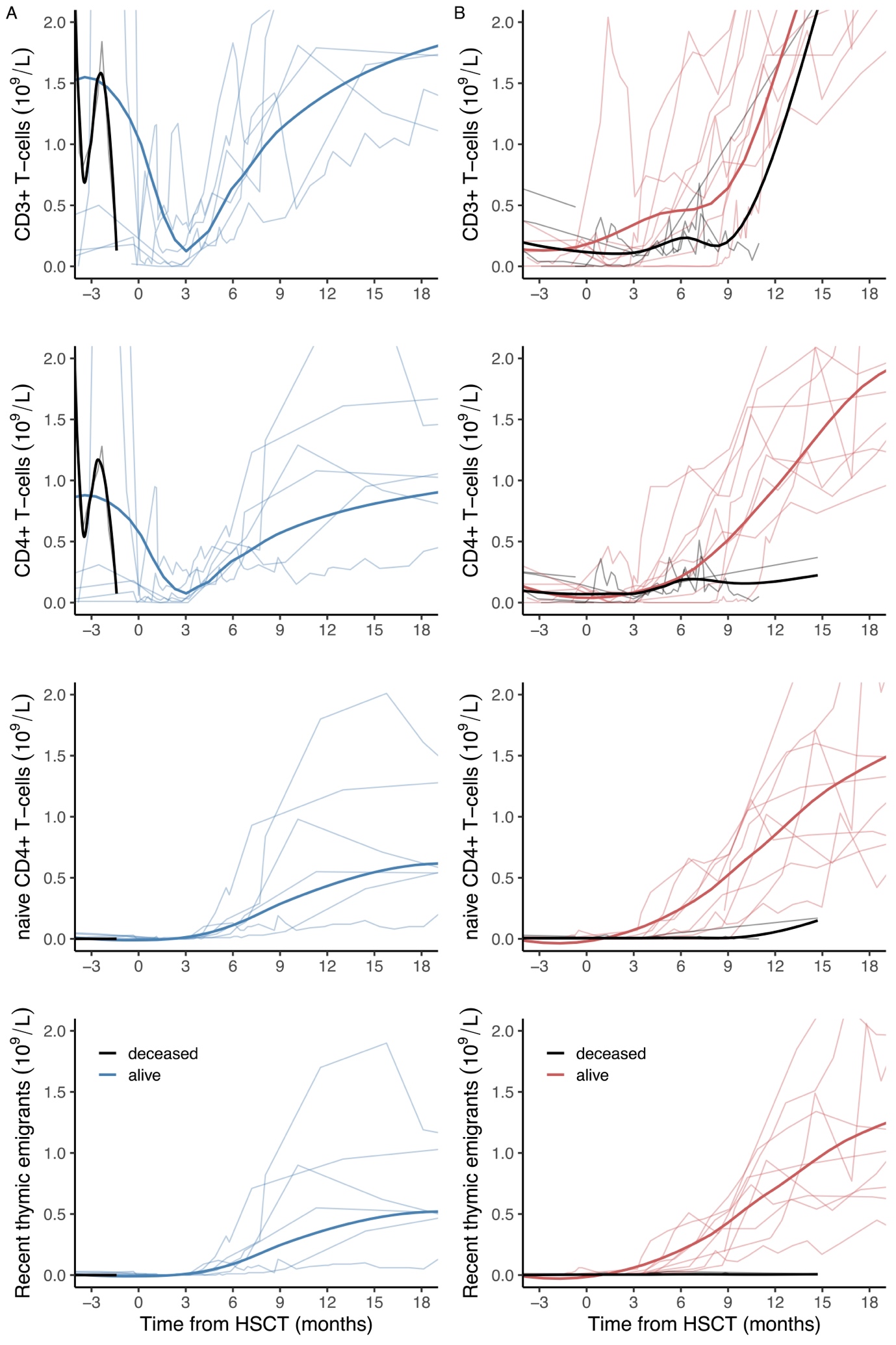


Figure 3S. The course of CD3+, CD4+, naïve CD4+ T-cell and recent thymic emigrant counts before and after hematopoietic stem cell transplantation in the newborn screening (A) and the clinical group (B). The survivors in each group are shown in the respective color, the deceased patients in black. HSCT – hematopoietic stem cell transplantation.


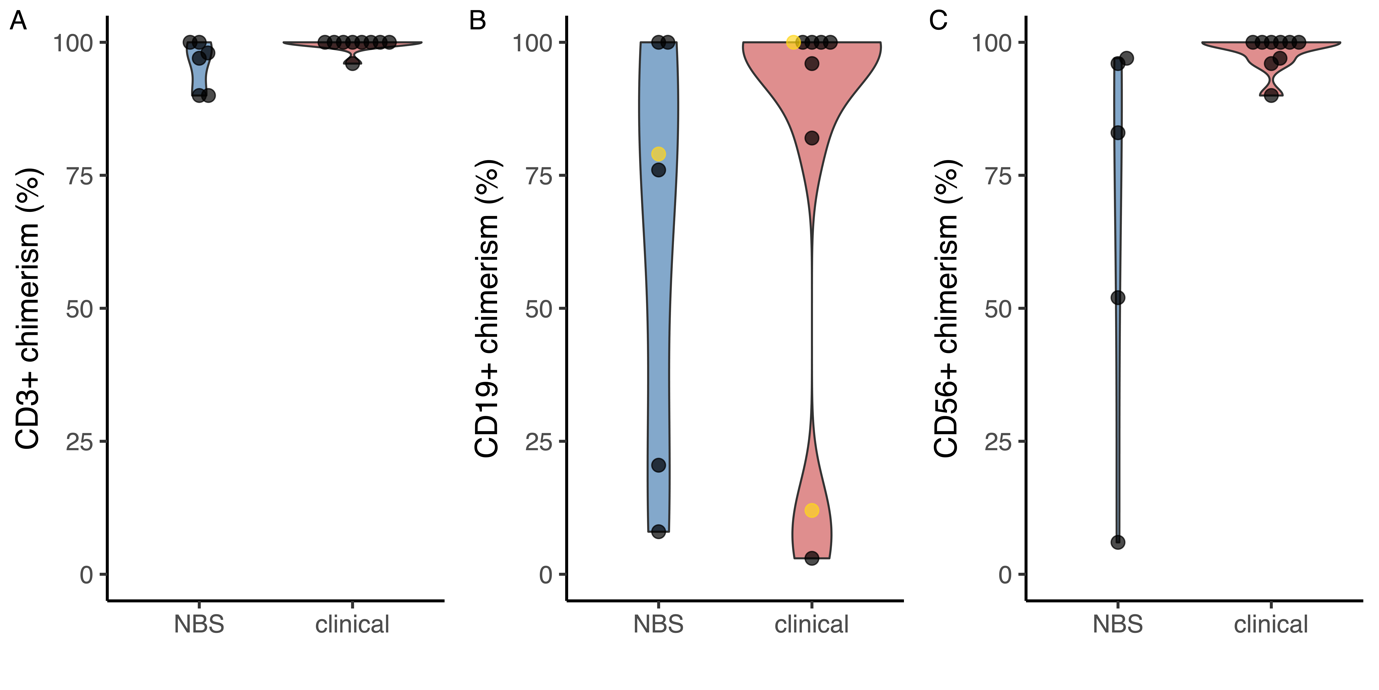


Figure 4S. CD3+ (A), CD19+ (B) and CD56+ (C) donor chimerism in survivors on last follow-up in the newborn screening groups (n=6) and in the clinical (n=9, one patient with no data) groups. Patients still on immunoglobulin substitution are marked with yellow. NBS – newborn screening.
